# Asymmetrical ligand-induced cross-regulation of chemokine (C-X-C motif) receptor 4 by α_1_-adrenergic receptors at the heteromeric receptor complex

**DOI:** 10.1038/s41598-018-21096-4

**Published:** 2018-02-09

**Authors:** Xianlong Gao, Lauren J. Albee, Brian F. Volkman, Vadim Gaponenko, Matthias Majetschak

**Affiliations:** 1 Burn and Shock Trauma Research Institute, Department of Surgery, Loyola University Chicago Stritch School of Medicine, Maywood, Illinois 60153 USA; 2 Department of Biochemistry, Medical College of Wisconsin, Milwaukee, Wisconsin 53226 USA; 3 Department of Biochemistry and Molecular Genetics, University of Illinois at Chicago, Chicago, Illinois 60607 USA; 4 Department of Molecular Pharmacology and Therapeutics, Loyola University Chicago Stritch School of Medicine, Maywood, Illinois 60153 USA

## Abstract

Recently, we reported that chemokine (C-X-C motif) receptor (CXCR)4 and atypical chemokine receptor 3 regulate α_1_-adrenergic receptors (α_1_-AR) through the formation of hetero-oligomeric complexes. Whether α_1_-ARs also regulate chemokine receptor function within such heteromeric receptor complexes is unknown. We observed that activation of α_1b_-AR within the α_1b_-AR:CXCR4 heteromeric complex leads to cross-recruitment of β-arrestin2 to CXCR4, which could not be inhibited with AMD3100. Activation of CXCR4 did not cross-recruit β-arrestin2 to α_1b_-AR. A peptide analogue of transmembrane domain 2 of CXCR4 interfered with α_1b_-AR:CXCR4 heteromerization and inhibited α_1b_-AR-mediated β-arrestin2 cross-recruitment. Phenylephrine (PE) induced internalization of CXCR4 in HEK293 cells co-expressing CXCR4 and α_1b_-AR and of endogenous CXCR4 in human vascular smooth muscle cells (hVSMC). The latter was detectable despite blockade of CXCR4 with the neutralizing antibody 12G5. hVSMC migrated towards CXCL12 and PE, but not towards a combination of CXCL12 and PE. PE inhibited CXCL12-induced chemotaxis of hVSMC (IC_50_: 77 ± 30 nM). Phentolamine cross-inhibited CXCL12-induced chemotaxis of hVSMC, whereas AMD3100 did not cross-inhibit PE-induced chemotaxis. These data provide evidence for asymmetrical cross-regulation of CXCR4 by α_1_-adrenergic receptors within the heteromeric receptor complex. Our findings provide mechanistic insights into the function of α_1_-AR:CXCR4 heteromers and suggest alternative approaches to modulate CXCR4 in disease conditions.

## Introduction

Chemokine (C-X-C motif) receptor (CXCR) 4 fulfills pleiotropic roles in the immune system and is regarded as an important regulator of directed cell migration^1^. Based on the involvement of CXCR4 in various disease processes, such as cancer metastases, HIV infection or inflammatory diseases, multiple drugs targeting CXCR4 are currently under development and the CXCR4 antagonist AMD3100 is already approved by the Federal Drug Administration to mobilize hematopoietic stem cells in cancer patients^1–5^. CXCR4 is a typical G protein-coupled receptor (GPCR)^1^. Upon binding to its cognate ligand CXCL12, CXCR4 couples to guanine nucleotide-binding protein α_i_ (Gα_i_) and also recruits β-arrestin 1/2 to the receptor, leading to termination of G protein-mediated signaling, receptor internalization and activation of G protein-independent signaling pathways^1,6^. Several lines of evidence suggest that CXCR4 can form hetero-oligomeric complexes with other GPCRs, such as chemokine (C-C motif) receptor (CCR)2, CCR5, CXCR3, atypical chemokine receptor (ACKR) 3, chemerin receptor 23, β_2_-adrenergic receptor (AR), δ-opioid receptor or cannabinoid receptor 2, which is thought to alter the pharmacological properties of the interacting receptor partners^7–14^. Recently, we provided evidence that hetero-oligomeric complexes between CXCR4, ACKR3 and α_1_-AR are constitutively expressed on the cell surface of human vascular smooth muscle cells (hVSMC), which appear to be essential for normal α_1_-AR function and through which the chemokine receptors upon agonist stimulation diametrically regulate α_1_-AR signaling and function^15–17^. The effects of such hetero-oligomerization on CXCR4-mediated signaling and function, however, remain unknown. Here, we provide evidence for asymmetrical ligand-induced cross-regulation of CXCR4 by α_1_-AR within heteromeric CXCR4:α_1_-AR complexes in hVSMC.

## Results and Discussion

### α_1b_-AR within the CXCR4:α_1b_-AR heteromeric complex cross-recruits β-arrestin 2 to CXCR4

**Figure 1:**
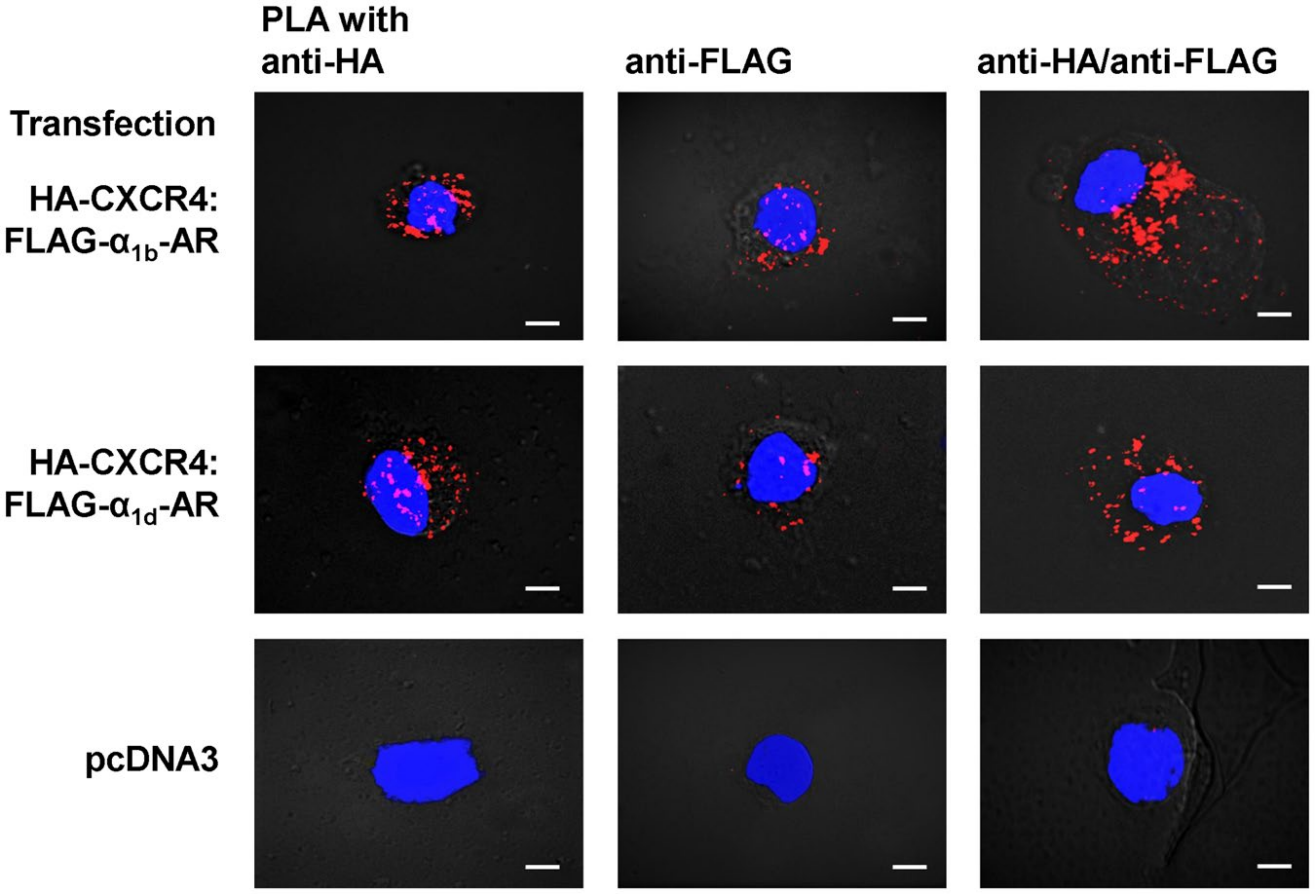
HA-CXCR4 forms heteromeric complexes with FLAG-α_1b/d_-AR in HEK293T cells. Typical PLA images for the detection of individual receptors (left and center) and receptor-receptor interactions (right) in HEK293T cells transfected with DNA encoding HA- and/or FLAG-tagged receptors, as indicated. PLA was performed with anti-HA and anti-FLAG. pcDNA3: control, cells were transfected with empty vector. Images show merged PLA/4′,6-diamidino-2-phenylindole dihydrochloride (DAPI) signals. Scale bars: 10 μm. Images are representative of n = 3 independent experiments.

**Figure 2:**
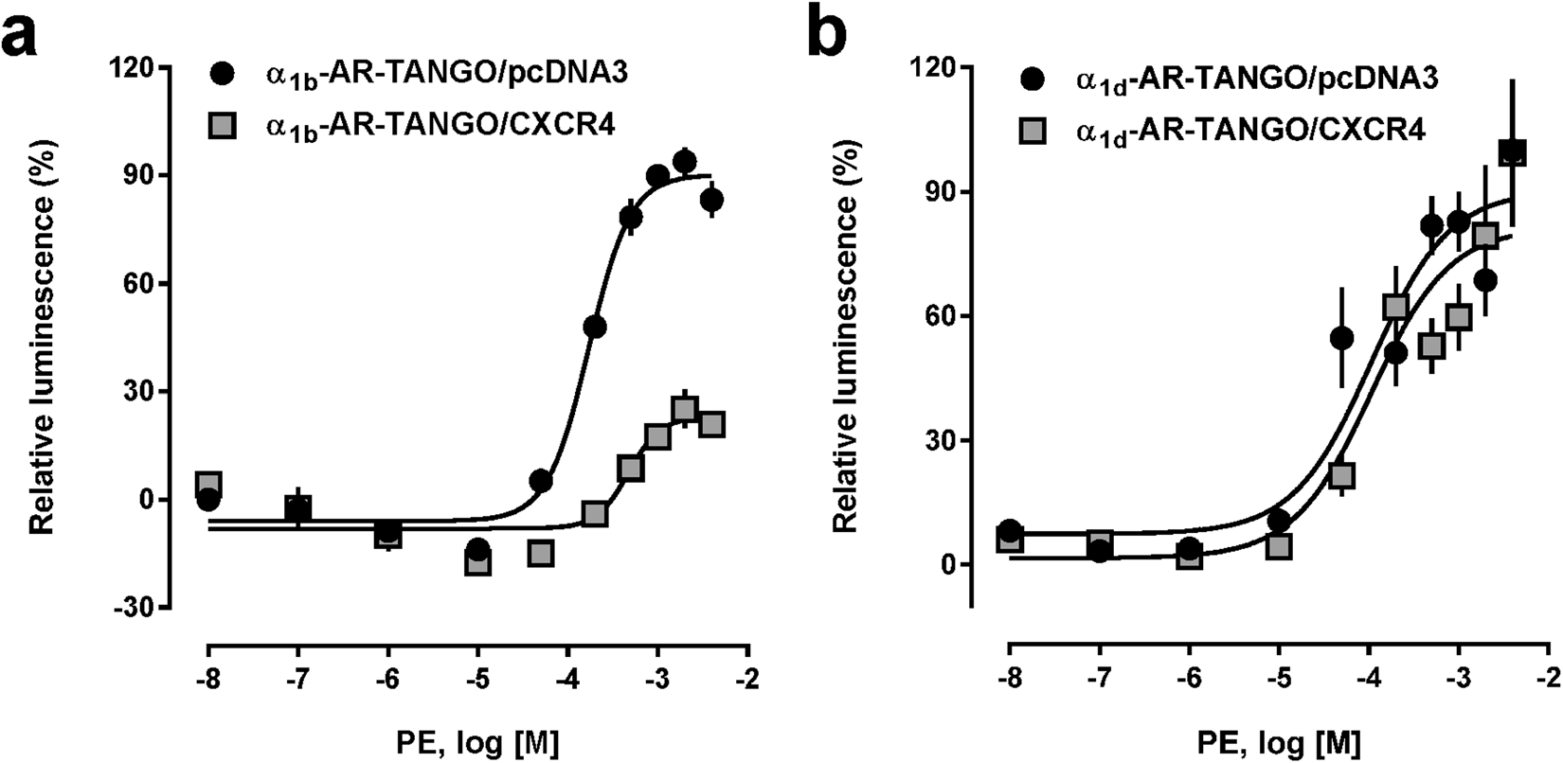
β-arrestin 2 recruitment to α_1b/d_-AR in the presence and absence of CXCR4. HTLA cells were co-transfected with 0.75 μg DNA encoding FLAG-α_1b_-AR-TANGO (**a**) or FLAG-α_1d_-AR-TANGO (**b**) plus 0.75 μg pcDNA3 or HA-CXCR4. PE: phenylephrine. (**a)** β-arrestin 2 recruitment assay (PRESTO-Tango). Black circles: cells transfected with FLAG-α_1b_-AR-TANGO/pcDNA3; grey squares: cells transfected with FLAG-α_1b_-AR-TANGO/HA-CXCR4. N = 3 independent experiments. **(b)** β-arrestin 2 recruitment assay (PRESTO-Tango). Black circles: cells transfected with FLAG-α_1d_-AR-TANGO/pcDNA3; grey squares: cells transfected with FLAG-α_1d_-AR-TANGO/HA-CXCR4. N = 3 independent experiments.

To reconfirm that recombinant CXCR4 and α_1b/d_-AR form heteromeric complexes, we co-transfected HEK293T cells with plasmids encoding human influenza hemagglutinin (HA)-tagged CXCR4 (HA-CXCR4) and with plasmids encoding FLAG-tagged α_1b_-AR (FLAG-α_1b_-AR) or FLAG-α_1d_-AR. We then performed proximity ligation assays (PLA) to visualize individual receptors and receptor-receptor interactions at single molecule resolution with anti-HA and anti-FLAG antibodies. Consistent with our previous observations^15–17^, we observed positive signals for interactions between HA-CXCR4 and FLAG-α_1b/d_-AR (Fig. 1). Next, we utilized the PRESTO-Tango cell system, which permits monitoring of β-arrestin 2 recruitment to a specific receptor^18^, to assess whether the CXCR4:α_1b/d_-AR interaction affects β-arrestin 2 recruitment upon agonist activation of each receptor partner. We first co-expressed FLAG-α_1b/d_-AR-Tango with pcDNA3 or HA-CXCR4, confirmed HA-CXCR4 expression and comparable FLAG-α_1b/d_-AR-Tango expression by flow cytometry (Supplementary Fig. S1) and determined the dose-response for β-arrestin 2 recruitment upon stimulation with the selective α_1_-AR agonist phenylephrine (PE). As shown in Fig. 2a, the EC_50_ for PE-induced β-arrestin 2 recruitment to α_1b_-AR-Tango was 172 μM (95% confidence interval (CI): 143–202 μM) and the top plateau 90 ± 2 relative luminescence units (RLU)% in cells transfected with α_1b_-AR/pcDNA3. In cells co-transfected with α_1b_-AR-Tango/CXCR4, potency and efficacy of PE to recruit β-arrestin 2 to α_1b_-AR were significantly reduced, as compared to cells co-transfected with α_1b_-AR/pcDNA3 (EC_50_: 475 (95%CI: 290–807) μM, p = 0.0001; top plateau: 23 ± 3 RLU, p < 0.0001). Because there were no differences in PE-induced β-arrestin 2 recruitment to α_1d_-AR in cells co-transfected with α_1d_-AR-Tango and pcDNA3 or CXCR4 (Fig. 2b), the observed effects appear to be specific for the α_1b_-AR:CXCR4 interaction.

**Figure 3:**
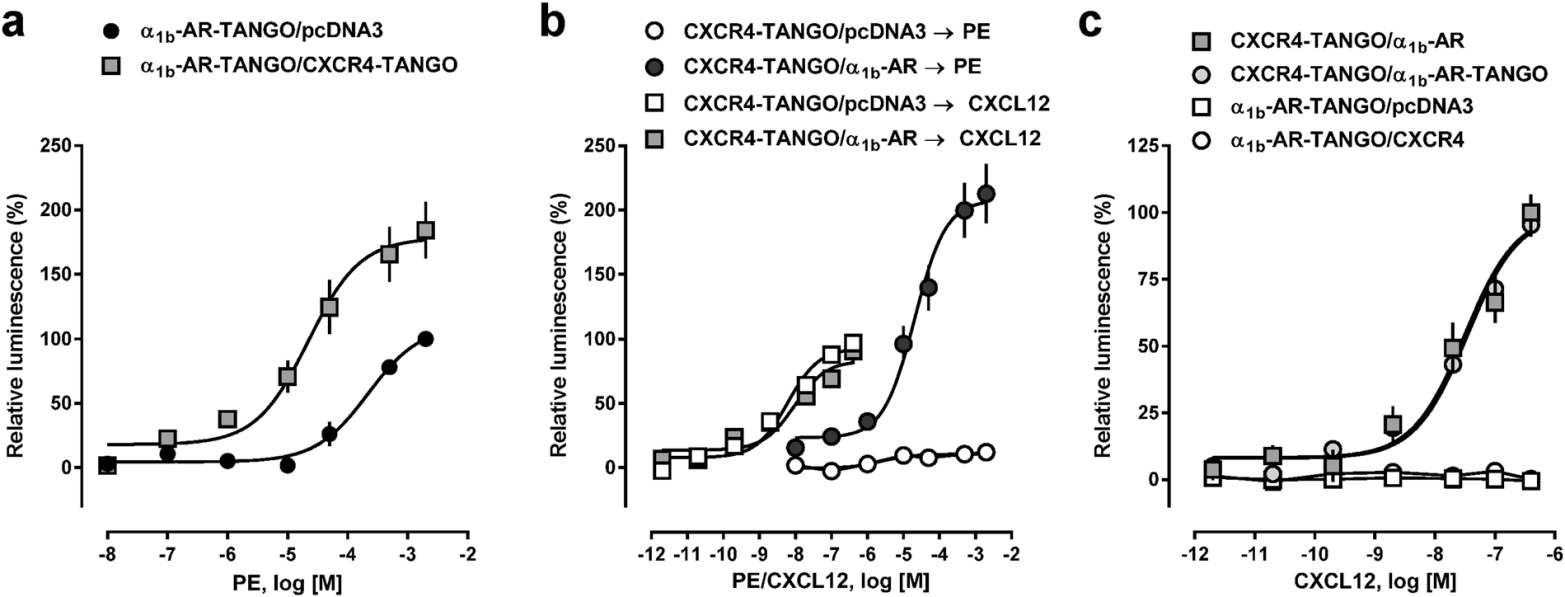
Activation of α_1b_-AR leads to cross-recruitment of β-arrestin 2 to CXCR4. HTLA cells were co-transfected with FLAG-α_1b_-AR-TANGO plus pcDNA or Myc-CXCR4-TANGO (**a**), FLAG-CXCR4-TANGO plus pcDNA or HA-α_1b_-AR (**b**) or with FLAG-CXCR4-TANGO plus HA-α_1b_-AR or Myc-α_1b_-AR-TANGO or with FLAG-α_1b_-AR-TANGO plus pcDNA3 or HA-CXCR4 (**c**) (0.75 μg DNA each). PE: phenylephrine. (**a)** β-arrestin 2 recruitment assay (PRESTO-Tango). Black circles: cells transfected with FLAG-α_1b_-AR-TANGO/pcDNA3; grey squares: cells transfected with FLAG-α_1b_-AR-TANGO/CXCR4-TANGO. N = 3 independent experiments. (**b)** β-arrestin 2 recruitment assay (PRESTO-Tango). Open symbols: cells transfected with FLAG-CXCR4/pcDNA3. Grey symbols: cells transfected with FLAG-CXCR4/α_1b_-AR. Cells were stimulated with phenylephrine (PE, circles) or CXCL12 (squares). N = 4 independent experiments. (**c)** β-arrestin 2 recruitment assay (PRESTO-Tango). Grey squares: cells transfected with FLAG-CXCR4-TANGO/α_1b_-AR. Grey circles: cells transfected with FLAG-CXCR4-TANGO/α_1b_-AR-TANGO. Open squares: cells transfected with FLAG-CXCR4-TANGO/pcDNA3. Open circles: cells transfected with α_1b_-AR-TANGO/CXCR4. N = 3 independent experiments.

While reduced β-arrestin 2 recruitment to α_1b_-AR-Tango in the presence of CXCR4 could be explained by inhibition of α_1b_-AR-Tango by CXCR4 through allosteric interactions within the heteromeric receptor complex, it could also be explained by cross-recruitment of β-arrestin 2 to CXCR4 upon α_1b_-AR-Tango activation. To explore this possibility, we then co-transfected cells with α_1b_-AR-Tango and pcDNA3 or with α_1b_-AR-Tango and CXCR4-Tango. Despite comparable α_1b_-AR-Tango expression under both conditions (Supplementary Fig. S2), we detected that the efficacy and potency of PE to induce β-arrestin 2 recruitment was significantly increased in cells co-transfected with α_1b_-AR-Tango and CXCR4-Tango, as compared with cells co-transfected with α_1b_-AR-Tango and pcDNA3 (α_1b_-AR-Tango/pcDNA3: EC_50_ (95%CI) 218 (130–276) μM, top plateau: 110 ± 7 RLU%; α_1b_-AR-Tango/CXCR4-Tango: EC_50_ (95%CI) 22 (9–53) μM - p = 0.0027, top plateau: 178 ± 11 RLU% - p = 0.03) (Fig. 3a).

As these data suggested that activation of α_1b_-AR recruits β-arrestin 2 to α_1b_-AR and CXCR4, we then expressed CXCR4-Tango in combination with α_1b_-AR or pcDNA, confirmed comparable CXCR4-Tango expression by flow cytometry (Supplementary Fig. S2) and measured β-arrestin 2 recruitment to CXCR4 upon PE stimulation. As shown in Fig. 3b, PE did not recruit β-arrestin 2 to CXCR4-Tango when co-expressed with pcDNA3, but recruited β-arrestin 2 to CXCR4-Tango when co-expressed with α_1b_-AR. Interestingly, the potency of PE for β-arrestin 2 recruitment to CXCR4-Tango under these conditions (EC_50_: 20.5 (95%CI: 9–45) μM) was ∼10-*fold* higher than the potency of PE for β-arrestin 2 recruitment to α_1b_-AR-Tango co-transfected with pcDNA3 and ∼20-*fold* higher than the potency of PE for β-arrestin 2 recruitment to α_1b_-AR-Tango co-transfected with CXCR4. Similarly, the efficacy of PE to recruit β-arrestin 2 to CXCR4-Tango under these conditions was 2-*fold* higher than the efficacy of the cognate CXCR4 agonist CXCL12 (Fig. 3b). The presence of α_1b_-AR, however, did not affect potency and efficacy of CXCL12 to recruit β-arrestin 2 to CXCR4-Tango (Fig. 3b). Similarly, there were no differences in CXCL12-induced β-arrestin 2 recruitment to CXCR4-Tango when comparable levels of CXCR4-Tango (Supplementary Fig. S2) were co-expressed with α_1b_-AR or α_1b_-AR-Tango in parallel experiments (Fig. 3c). In contrast to PE, CXCL12 failed to cross-recruit β-arrestin 2 to α_1b_-AR-Tango in cells co-expressing CXCR4 and α_1b_-AR-Tango (Fig. 3c).

**Figure 4:**
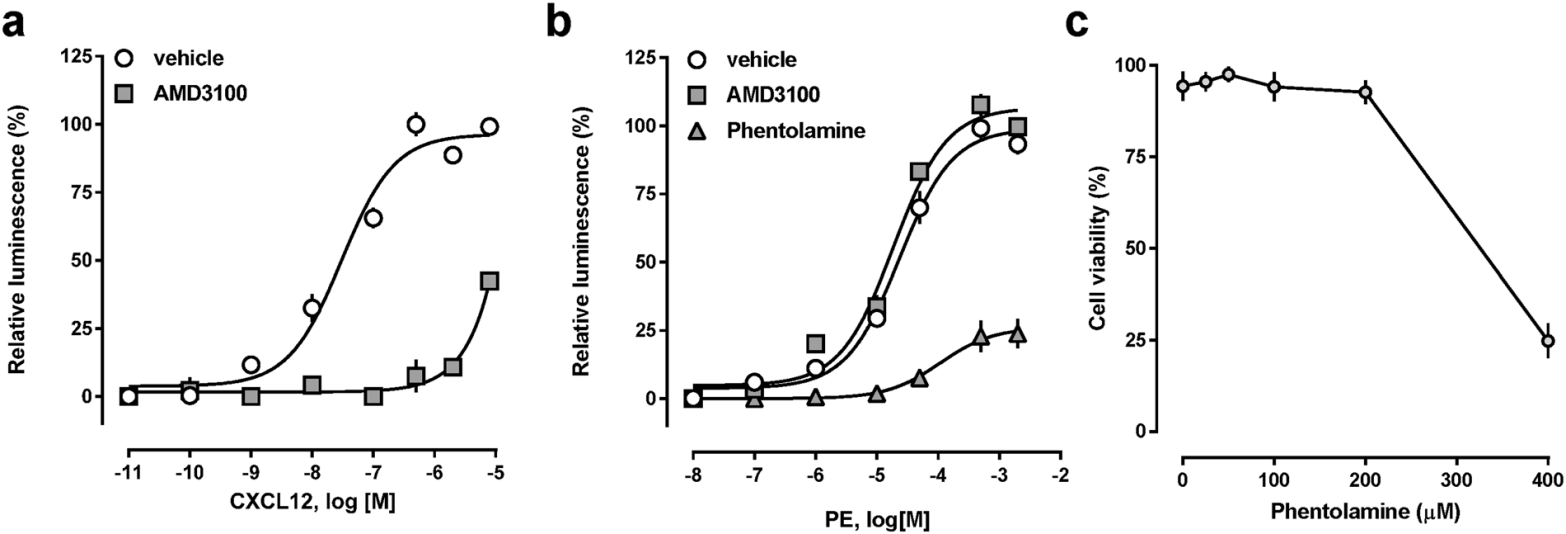
Effects of receptor antagonists on cross-recruitment of β-arrestin 2 to CXCR4 upon α_1b_-AR activation. PE: phenylephrine. (**a)** β-arrestin 2 recruitment assay (PRESTO-Tango). Cells were transfected with FLAG-CXCR4-TANGO/pcDNA3 and stimulated with CXCL12 in the presence of vehicle (open circles) or AMD3100 (10 μM). N = 3 independent experiments. **(b)** β-arrestin 2 recruitment assay (PRESTO-Tango). Cells were transfected with FLAG-CXCR4-TANGO/α_1b_-AR and stimulated with PE after pre-treatment (15 min, 37 °C) with vehicle (open circles), AMD3100 (10 μM) or phentolamine (100 μM). N = 3 independent experiments. (**c)** Cells were treated with various concentrations of phentolamine, as in b., and cell viability was determined by trypan blue exclusion. N = 3 independent experiments.

To further consolidate that agonist stimulation of α_1b_-AR leads to cross-recruitment of β-arrestin 2 to CXCR4, we then tested the effects of the selective α_1_-AR antagonist phentolamine and of the selective CXCR4 antagonist AMD3100 on PE-induced β-arrestin 2 cross-recruitment to CXCR4-Tango. AMD3100 inhibited CXCL12 induced β-arrestin 2 recruitment to CXCR4-Tango (Fig. 4a). While AMD3100 did not inhibit PE-induced β-arrestin 2 recruitment to CXCR4-Tango in cells co-expressing CXCR4-Tango and α_1b_-AR, phentolamine inhibited PE-induced β-arrestin 2 recruitment under these conditions (Fig. 4b). Measurements of cell viability demonstrated that phentolamine was not cytotoxic at the concentration that was used in the β-arrestin 2 recruitment assays (Fig. 4c).

**Figure 5:**
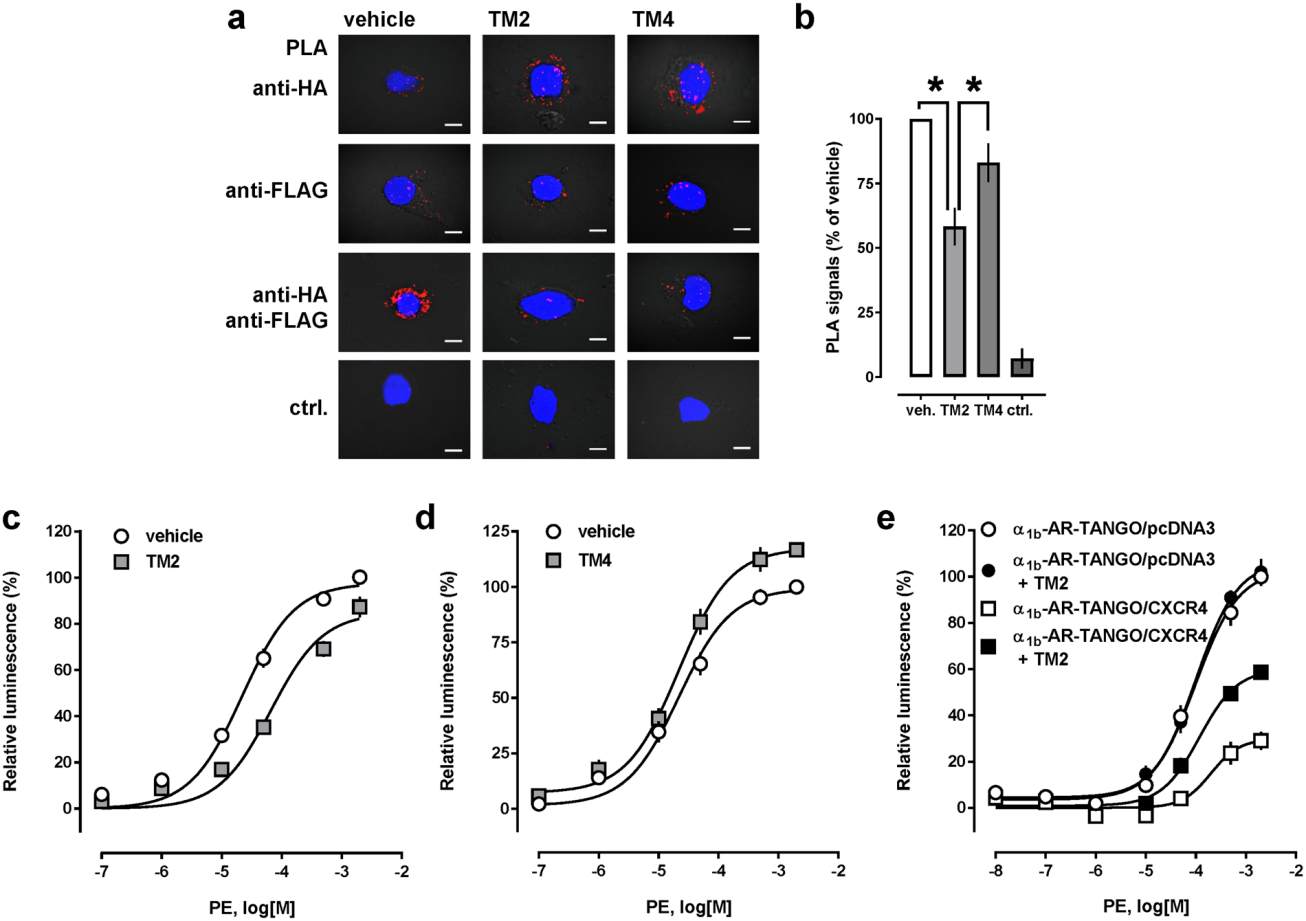
Effects of peptide analogues of transmembrane domains of CXCR4 on cross-recruitment of β-arrestin 2 to CXCR4 upon α_1b_-AR activation. (**a**) Cells were co-transfected with HA-α_1b_-AR and FLAG-CXCR4, treated with vehicle or peptide analogues of transmembrane domain (TM) 2 and TM4 (50 μM, 15 min, 37 °C), and used for PLA. Typical PLA images for the detection of (from top to bottom) HA-α_1b_-AR, FLAG-CXCR4 and interactions between both receptors are shown. Ctrl.: control, cells transfected with empty vector and stained for interactions between HA-α_1b_-AR and FLAG-CXCR4. Scale bars: 10 μm. (**b**) Quantification of PLA signals for FLAG-CXCR4-TANGO/HA-α_1b_-AR interactions from n = 3 independent experiments, as in a. PLA signals/cell are expressed as % of vehicle treated cells. *p < 0.05 vs. cells incubated with TM2. (**c**) β-arrestin 2 recruitment assay (PRESTO-Tango). Cells were transfected with FLAG-CXCR4-TANGO/α_1b_-AR and stimulated with PE after pre-treatment (15 min, 37 °C) with vehicle (open circles) or the TM2 (50 μM) peptide (grey squares). N = 5 independent experiments. (**d**) β-arrestin 2 recruitment assay (PRESTO-Tango). Cells were transfected with FLAG-CXCR4-TANGO/α_1b_-AR and stimulated with PE after pre-treatment (15 min, 37 °C) with vehicle (open circles) or the TM4 (50 μM) peptide (grey squares). N = 4 independent experiments. (**e**) β-arrestin 2 recruitment assay (PRESTO-Tango). Cells were transfected as indicated and stimulated with PE after pre-treatment (15 min, 37 °C) with vehicle (open symbols) or the TM2 (50 μM) peptide (black symbols). N = 3 independent experiments.

Peptide analogues of transmembrane domains (TM) of GPCRs have previously been shown to interfere with receptor heteromerization and function and we reported that a peptide analogue of transmembrane domain 2 (TM2) of CXCR4 selectively interferes with CXCR4:α_1B_-AR heteromerization in hVSMC (15–17). Thus, we tested whether the TM2 peptide analogue would also disrupt recombinant CXCR4:α_1b_-AR heteromeric complexes and affect PE-induced β-arrestin 2 cross-recruitment to CXCR4. We used a TM4 peptide analogue of CXCR4 as a control peptide because we observed previously that this peptide analogue did not disrupt heteromerization between CXCR4:α_1B_-AR^17^. As shown in Fig. 5a,b, the TM2 peptide analogue significantly reduced PLA signals corresponding to CXCR4-Tango:α_1b_-AR interactions, as compared with vehicle-treated cells and cells treated with the TM4 peptide analogue. The TM4 peptide analogue did not significantly reduce PLA signals for CXCR4-Tango:α_1b_-AR interactions. In parallel experiments with cells co-expressing CXCR4-Tango and α_1b_-AR, we detected that the TM2 peptide reduced the potency of PE for β-arrestin 2 recruitment to CXCR4 3-*fold*, as compared with cells treated with vehicle (EC_50_ (95%CI): vehicle − 22^17–28^ μM; TM2 − 66 (47–93) μM, p < 0.001) (Fig. 5c). In contrast, the TM4 peptide analogue did not inhibit PE-induced β-arrestin 2 recruitment to CXCR4, when compared with vehicle treated cells in parallel experiments (Fig. 5d). While the TM2 peptide analogue did not interfere with PE-induced β-arrestin 2 recruitment in cells expressing α_1b_-AR-Tango alone, it significantly increased the efficacy of PE to recruit β-arrestin 2 in cells co-expressing α_1b_-AR-Tango and CXCR4 (top plateau: vehicle−29.8 ± 4.2%; TM2 − 60 ± 3.8%, p = 0.0101) (Fig. 5e). These findings are consistent with the assumption that cross-recruitment of β-arrestin 2 to CXCR4 upon α_1b_-AR activation depends on the formation of α_1b_-AR:CXCR4 heteromers.

### Recombinant CXCR4 internalizes upon agonist binding to α_1b_-AR

**Figure 6:**
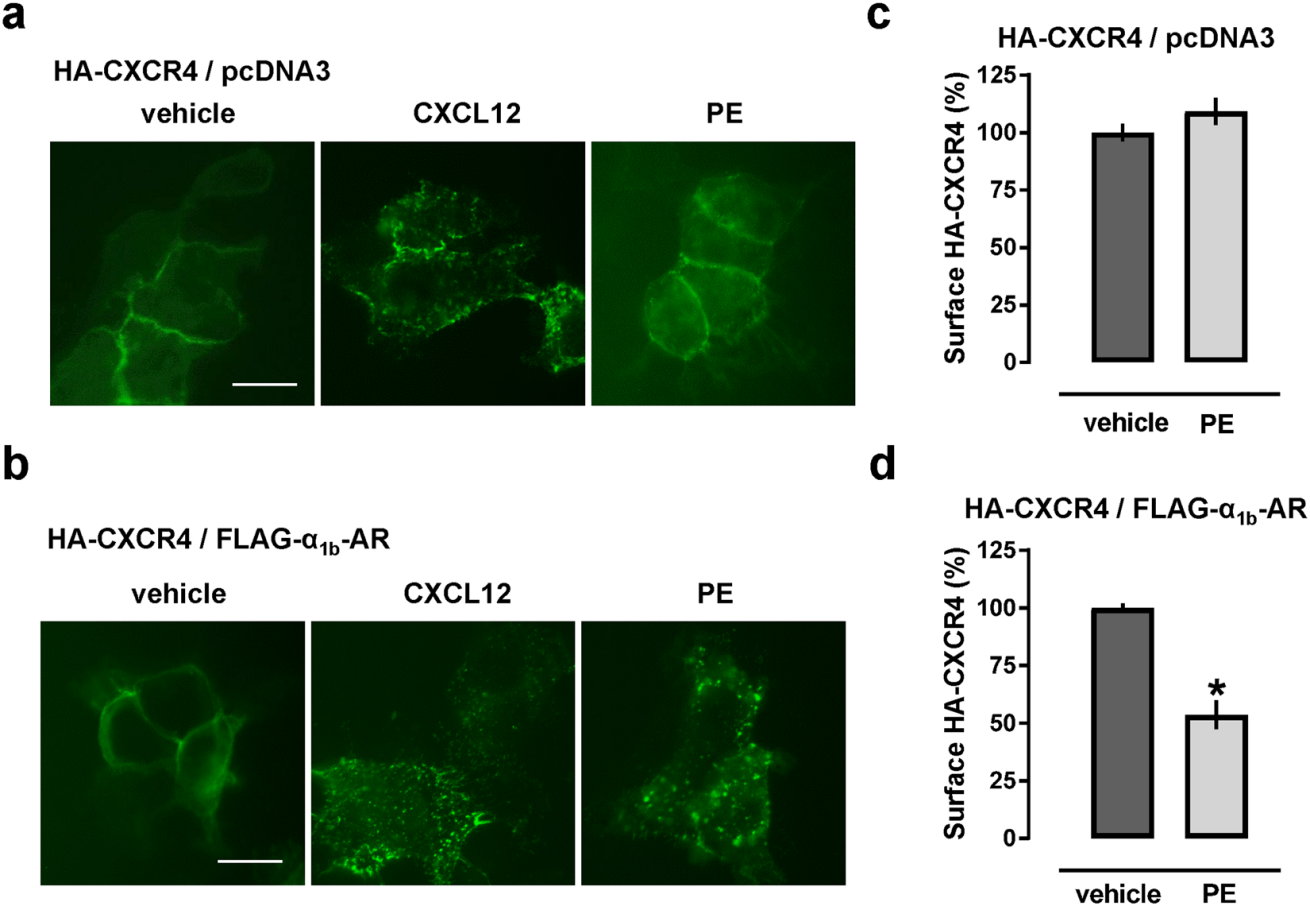
α_1b_-AR activation within the CXCR4:α1b-AR heteromer leads to internalization of CXCR4 in HEK293T cells. (**a**,**b**) After labeling HA-CXCR4 with FITC-conjugated anti-HA at 4 °C, HEK293T cells were treated with CXCL12 (200 nM) or phenylephrine (PE, 200 μM) for 1 hour at room temperature. Images are representative of n = 3 independent experiments. Scale bar: 20 μm. HEK293T cells were transfected with HA-CXCR4/pcDNA3 (**a**) or with HA-CXCR4/FLAG-α_1b_-AR (**b**). (**c,d**) Quantification of HA-CXCR4 cell surface expression by flow cytometry with FITC-anti-HA in cells after stimulation with PE, as in a/b. Cells were transfected with HA-CXCR4/pcDNA3 (**c**) or HA-CXCR4/α_1b_-AR (**d**). Surface HA-CXCR4 expression is expressed as % fluorescence intensity of cells treated with vehicle. *p < 0.05 vs. vehicle. N = 4 independent experiments.

Because β-arrestin recruitment to CXCR4 upon CXCL12 binding leads to internalization of the receptor, we then studied by immunofluorescence microscopy whether activation of α_1b_-AR would also result in the internalization of CXCR4 in cells co-expressing both recombinant receptors. After transfection with HA-CXCR4 plus pcDNA3 or α_1b_-AR, HEK293T cells were labeled with FITC-conjugated anti-HA at 4 °C, followed by stimulation with vehicle, CXCL12 or PE at room temperature. In cells co-expressing HA-CXCR4 and pcDNA3 (Fig. 6a), the fluorescence signal for FITC-labeled anti-HA was localized to the cell membrane after vehicle and PE treatment (left and right). After treatment with CXCL12, punctate areas of fluorescence were detectable, indicating internalization of HA-CXCR4 (center). In cells co-expressing HA-CXCR4 and α_1b_-AR (Fig. 6b), CXCL12 and PE treatment resulted in punctate areas of fluorescence, suggesting that HA-CXCR4 internalizes upon activation of α_1b_-AR. Measurements of cell surface HA-CXCR4 expression levels by flow cytometry confirmed these observations and showed that PE did not affect HA-CXCR4 expression levels in cells co-expressing HA-CXCR4/pcDNA3 (Fig. 6c), but reduced HA-CXCR4 expression levels in cells co-expressing HA-CXCR4/α_1b_-AR (Fig. 6d).

### CXCR4 in human vascular smooth muscle cells internalizes upon agonist binding to α_1_-AR

**Figure 7:**
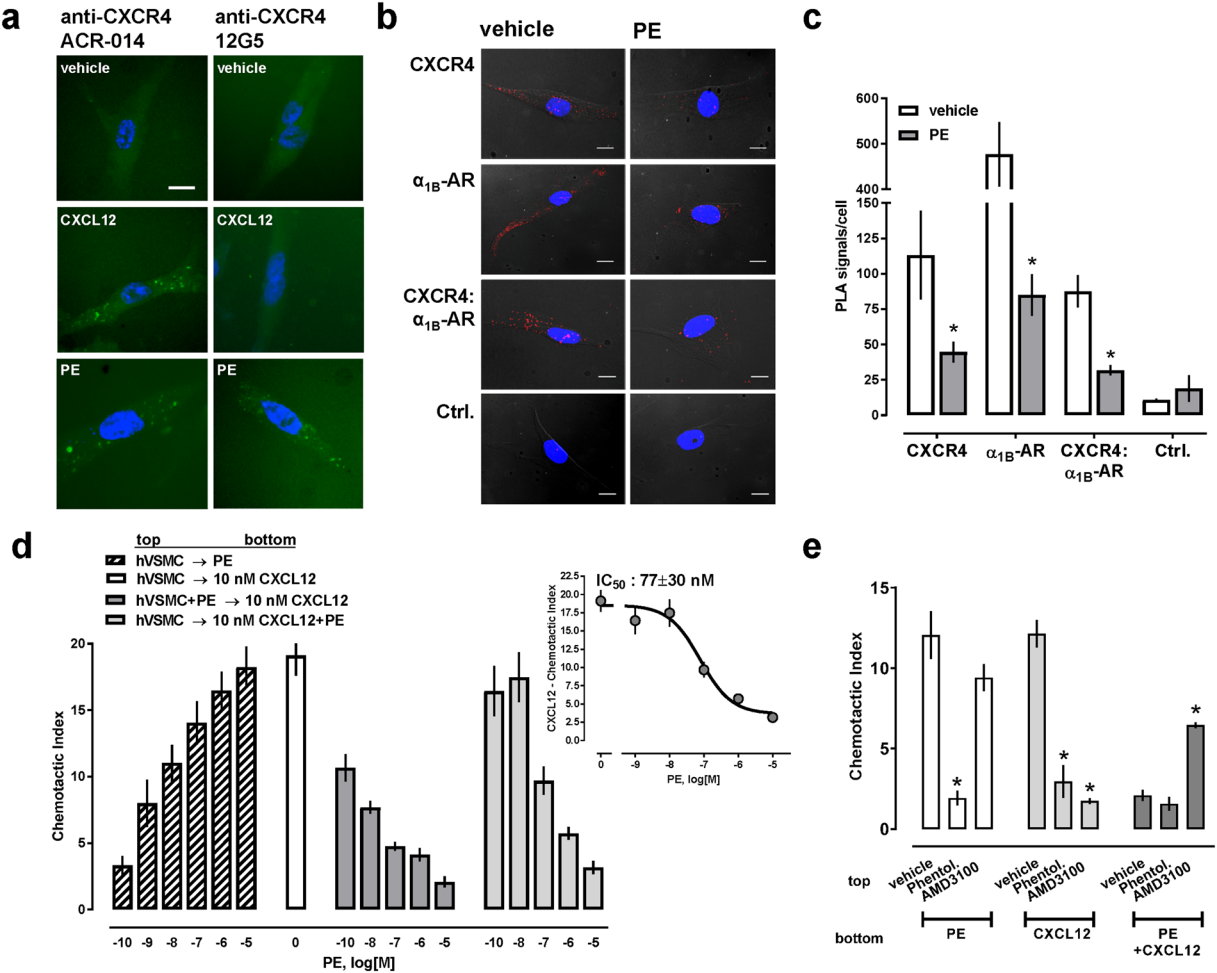
α_1_-AR regulate internalization of CXCR4 in human vascular smooth muscle cells and CXCR4-mediated chemotaxis. (**a**) hVSMC were labeled for 1 hour at 4 °C with rabbit anti-CXCR4 (ACR-014, left) or FITC-conjugated-anti-CXCR4 (12G5, right), and then treated with vehicle, 100 nM CXCL12 or 10 μM PE for 20 min at 37 °C. Cells stained with anti-CXCR4 (ACR-014) were permeabilized with TX-100 and stained with Alexa 488-conjugated anti-rabbit. Fluorescence microscopy images are representative of n = 3 independent experiments. Scale bar: 20 μm. (**b**) Representative PLA images for the detection of individual receptors and receptor-receptor interactions in hVSMC. Cells were treated with vehicle or PE (1μM) for 15 min at 37 °C. Ctrl.: Omission of one primary antibody. Images show merged PLA/4′,6-diamidino-2-phenylindole dihydrochloride signals. Scale bars = 10 μm. (**c**) Quantification of PLA signals, as in b. N = 3 with n = 10 images per condition and experiment. *p < 0.05 vs. ctrl. (**d**) Migration of hVSMC towards PE (hatched bars), CXCL12 (10 nM, open bar) and combinations of both (grey bars). Dark grey bars: migration of hVSMC in the presence of various concentrations of PE towards 10 nM CXCL12. Light grey bars: migration of hVSMC towards 10 nM CXCL12 plus various concentrations of PE (insert shows the dose-response curve). N = 4 independent experiments. (**e**) hVSMC were pre-treated with 10 μM phentolamine, 10 μM AMD3100, or vehicle for 15 min at room temperature, followed by migration towards 1 μM PE, 10 nM CXCL12, or both. N = 4 independent experiments. *p < 0.05 vs. vehicle.

To assess whether PE stimulation also induces internalization of endogenous CXCR4, we then incubated hVSMC with rabbit anti-CXCR4 ACR-014 at 4 °C, followed by incubation with vehicle, CXCL12 or PE at 37 °C. Cells were then fixed, permeabilized and incubated with Alexa488-conjugated goat anti-rabbit IgG. Fluorescence microscopy showed a diffuse pattern of fluorescence in vehicle treated cells (Fig. 7a, left column, top). In cells treated with CXCL12 and PE (Fig. 7a, left column, center and bottom), bright fluorescent punctate areas were visible. These observations are consistent with CXCR4 internalization and with the staining patterns that have previously been observed in similar fluorescence microscopy internalization studies of other GPCRs^19^. When these experiments were performed with FITC-labeled anti-CXCR4 12G5, an antibody known to inhibit CXCL12 binding to CXCR4^20^, fluorescent punctate formation did not occur after CXCL12 treatment, but persisted after PE treatment (Fig. 7a, right column). These findings are in line with our observation that blockade of CXCR4 with AMD3100 did not prevent β-arrestin 2 cross-recruitment upon α_1b_-AR activation (Fig. 4b). To confirm that PE treatment leads to internalization of CXCR4 in hVSMC, we then performed PLA to analyze expression levels of CXCR4, α_1B_-AR and CXCR4:α_1B_-AR heteromers after vehicle and PE treatment (Fig. 7b). The quantification of the PLA signals per cell from three independent experiments is shown in Fig. 7c. As compared with vehicle treated cells, PLA signals corresponding to CXCR4, α_1B_-AR and heteromeric CXCR4:α_1B_-AR complexes were significantly reduced after PE treatment. Together, our observations suggest that activation of α_1B_-AR within the α_1B_-AR:CXCR4 heteromer recruits β-arrestin to both receptor partners, leading to co-internalization of α_1B_-AR and CXCR4. Furthermore, our findings that AMD3100 and anti-CXCR4 12G5 did not prevent β-arrestin 2 recruitment and internalization of CXCR4 upon PE stimulation indicate that this receptor cross-talk can occur when CXCR4 is in its ligand-free and in an antagonist-bound configuration. This assumption is supported by the recent observation that chemically-induced fusion of CXCR4 to β-arrestin 1/2 leads to internalization of the receptor, independent of agonist binding to and G protein coupling through CXCR4^21^.

### Ligand binding to α_1_-AR inhibits CXCR4-mediated chemotaxis of human vascular smooth muscle cells

To assess consequences of ligand binding to α_1_-AR on CXCR4-mediated effects on cell function, we then studied chemotactic responses of hVSMC in trans-well migration assays. We reported previously that the dose-response profile of CXCL12 shows maximal chemotactic activity at concentrations between 10–100 nM in hVSMC under our experimental conditions^22^. As expected^23,24^, PE also dose-dependently induced chemotaxis in hVSMC under these conditions (Fig. 7d). Maximal chemotactic activity of PE was comparable with the chemotactic activity of CXCL12. When hVSMC were loaded onto the membrane in the presence of increasing concentrations of PE, migration towards 10 nM CXCL12 was significantly reduced. At a concentration of 10 nM PE, chemotaxis towards 10 nM CXCL12 was reduced by more than 50% and further increases in PE concentrations dose-dependently reduced the chemotactic index (CI) to 20% of the CI in the absence of PE (dark grey bars). When hVSMC migrated towards 10 nM CXCL12 plus increasing concentrations of PE, PE inhibited chemotaxis with an IC_50_ of 77 ± 30 nM (Fig. 7d, dark grey bars and inlay). These findings are in agreement with previous observations on the pharmacological behaviors of the CXCR4:δ-opioid receptor and CXCR4:cannabinoid receptor 2 heteromers, in which agonist co-stimulation of both receptor partners abolished chemotaxis towards CXCL12^13,14^.

As pharmacological cross-inhibition has previously been described for other GPCR heteromers, we then tested the effects of AMD3100 and phentolamine on the chemotactic responses of hVSMC to CXCL12, PE and a combination of both agonists. As in Fig. 7d, hVSMC migrated towards PE and CXCL12 and this response was inhibited when cells migrated towards both agonists (Fig. 7e). Phentolamine inhibited PE- and CXCL12-induced chemotaxis, but did not alter the chemotactic response to both agonists. In contrast, AMD3100 inhibited CXCL12-induced chemotaxis, but did not significantly affect PE-induced chemotaxis. AMD3100, however, restored a chemotactic response of hVSMC to the combination of PE and CXCL12. In combination with the effects of PE on β-arrestin 2 cross-recruitment to and co-internalization of CXCR4, these findings indicate asymmetrical cross-activation and cross-inhibition of CXCR4 within the heteromeric receptor complex by α_1_-AR ligands. Such a pharmacological behavior of the CXCR4:α_1_-AR heteromeric complex is similar to the signaling behavior of other GPCR heteromers, for which both ligand-induced symmetrical and asymmetrical cross-activation and cross-inhibition of various signaling read-outs have previously been described^25–28^. For example, symmetrical ligand induced cross-inhibition and cross-sensitization was observed for the angiotensin II receptor type 1:prostaglandin F2α receptor heteromer when aortic ring contraction was used as a functional read-out, whereas the effects of agonist stimulation on [^3^H]thymidine incorporation in vascular smooth muscle cells showed asymmetrical cross-inhibition, i.e. a prostaglandin F2α receptor antagonist inhibited angiotensin II-induced effects but an angiotensin II receptor type 1 antagonist did not affect prostaglandin F2α induced-effects. Similarly, agonist-induced cross-phosphorylation and cross-internalization was observed at the NK1 receptor:μ-opioid receptor heteromer^25^

It has been proposed previously that simultaneous agonist binding to both receptor partners can inhibit signaling of heteromeric receptor complexes, such as the CXCR4:cannabinoid receptor 2, CXCR4:δ-opioid receptor or μ-opioid receptor:CCR5 heteromers^13,14,29^. As the results from the present study point towards β-arrestin 2 cross-recruitment to AMD3100-bound CXCR4 upon α_1_-AR activation, a possible explanation for the observation that AMD3100 restored chemotaxis of hVSMC towards both agonists is that coupling of the heteromeric receptor complex to both Gα_i_ and Gα_q_ upon ligand binding is required to prevent PE-induced chemotaxis. The finding that phentolamine did not restore chemotaxis of hVSMC towards both agonists is not contradictive because phentolamine cross-inhibited chemotactic responses of hVSMC towards CXCL12 alone.

In conclusion, our findings provide new mechanistic insights into molecular signaling events at the CXCR4:α_1b_-AR hetero-oligomeric complex and evidence that α_1_-ARs regulate CXCR4-mediated functions in hVSMC. Our findings of asymmetrical agonist- and antagonist-induced cross-regulation of CXCR4 by α_1_-AR within the heteromeric receptor complex have several implications. While these data provide a mechanistic basis for interactions between the neuroendocrine and immune system, our findings also point towards α_1_-AR as new pharmacological target to modulate CXCR4 expression levels and CXCR4-mediated effects on cell migration in various disease processes, such as cancer metastasis or autoimmune diseases. These data further support the notion that the development of drugs which selectively target heteromeric receptor complexes could have novel and unique pharmacological properties and also imply that drugs with high selectivity for a specific GPCR can have unforeseen pharmacological effects through its actions on heteromerization receptor partners. Thus, the detailed elucidation of the molecular properties of heteromeric receptor complexes will provide new opportunities for drug development and may also help to better anticipate unwanted side-effect profiles of drugs targeting GPCRs.

## Methods

### Proteins, peptides and reagents

Phenylephrine, phentolamine and AMD3100 were purchased from Sigma Aldrich. CXCL12 was from Protein Foundry. The peptide analogs of transmembrane helix 2 (TM2) and TM4 of CXCR4 were as previously described^17^.

### Cells

Human vascular smooth muscle cells (hVSMC; primary aortic smooth muscle cells, ATCC-PCS-100-012) and HEK 293 T cells (ATCC-CRL-11268) were purchased from American Type Culture Collection. hVSMC were cultured in vascular basal media (ATCC PCS-100-030) supplemented with the vascular smooth muscle growth kit (ATCC PCS-100-042), 100 U/mL penicillin, 100 μg/mL streptomycin. hVSMCs were used between passages 2–5. HEK 293 T cells were cultured in high-glucose Dulbecco’s Modified’s Eagle Medium containing 10 mg/mL sodium pyruvate, 2 mM L-glutamine, 10% FBS, 100 U/mL penicillin, and 100 μg/mL streptomycin. The HTLA cell line, a HEK293 cell line stably expressing a tTA-dependent luciferase reporter and a β-arrestin2-TEV fusion gene^18^, was generously provided by the laboratory of Dr. Bryan Roth and maintained in high glucose Dulbecco’s Modified’s Eagle Medium supplemented with 10% FBS, 100 U/mL penicillin, 100 μg/mL streptomycin, 100 µg/mL hygromycin B, and 2 µg/mL puromycin. All cells were cultured in a humidified environment at 37 °C, 5% CO_2_.

### Flow cytometry

Flow cytometry after labeling cells with Phycoerythrin-conjugated anti-FLAG antibody (Biolegend, 637310), Alexa 647-conjugated anti-FLAG antibody (R&D, IC8529R), Alexa 488-conjugated anti-Myc antibody (R&D, IC3696G) or FITC-conjugated anti-HA antibody (Sigma-Aldrich, H7411) was used to quantify recombinant receptor expression levels in HEK293T cells, as described^15,30,31^. At least 10,000 cells/sample were recorded and analyzed with the FlowJo software (Tree Star).

### Proximity Ligation Assays (PLA)

PLA were performed as described in detail previously^15,16^. To visualize individual receptors, slides were incubated with mouse anti-FLAG M2 (Sigma-Aldrich, F1804), rabbit anti-HA (Abcam, Ab9110), rabbit anti-α_1B_-AR (Abcam Ab169523) or goat anti-CXCR4 (Abcam Ab1670) at 37 °C for 105 min in a humidifying chamber at 37 °C. Anti-α_1B_-AR and anti-CXCR4 have been validated for sufficient selectivity previously^16,31^. Slides were then washed and incubated (1 h, 37°C) with secondary species specific (rabbit/mouse/goat) antibodies conjugated with plus and minus Duolink II PLA probes (1:5), as appropriate. To visualize receptor-receptor interactions, slides were incubated with a combination of rabbit anti-α_1B_-AR (Abcam Ab169523) and goat anti-CXCR4 (Abcam Ab1670) or a combination of mouse anti-FLAG M2 and rabbit anti-HA. All antibodies, except anti-HA (dilution 1:1000), were used in dilutions of 1:500. Slides were then washed and incubated with secondary species specific antibodies conjugated with plus and minus Duolink II PLA probes (1:5) corresponding to the two primary antibodies. PLA signals were quantified using the Duolink Image Tool software (Sigma Aldrich). Comparisons and statistical analyses were performed only when PLA assays were performed on the same day in parallel experiments and fluorescence microscopy was performed with the identical settings. For each experiment and condition, 10 randomly selected non-overlapping vision fields were analyzed.

### Plasmids and transfections

FLAG-tagged Tango plasmids (CXCR4-TANGO, #66262; α_1b_-AR-TANGO, #66214; α_1d_-TANGO, #66215) were from Addgene deposited by the laboratory of Dr. Bryan Roth. Myc-tagged TANGO plasmids were generated by replacing the original FLAG-tag plasmids with Myc tag after PCR amplification with primers carrying the Myc tag sequence. HA- or FLAG-tagged CXCR4, α_1b_-AR or α_1d_-AR were generated by PCR amplification using corresponding TANGO plasmids as cDNAs with primers carrying Xho I and Xba I sites and inserted in pcDNA3 with an N-terminal FLAG or HA tag. All plasmids were verified by sequencing. HEK293T cells were transiently transfected with a single DNA or two DNAs that encode HA- or FLAG-tagged CXCR4, α_1b_-AR or α_1d_-AR or TANGO plasmids at the amounts indicated, using Lipofectamine 3000 (Thermo Scientific) as per manufacturer’s protocol. For single GPCR-encoding DNA transfection, empty vector pcDNA3 was added to maintain the total DNA amount per transfection constant.

### PRESTO-Tango β-arrestin recruitment assay

The PRESTO-Tango (parallel receptorome expression and screening via transcriptional output, with transcriptional activation following arrestin translocation) assay was performed as recently described^18^. HTLA cells (2.5 × 10^5^/well) were seeded in a 6-well plate and transfected with 750 ng of each of the Tango plasmids using Lipofectamine 3000 (Thermo Scientific). The following day, transfected HTLA cells (75,000 cells/well) were plated onto Poly-L-Lysine pre-coated 96-well microplates and allowed to attach to the plate surface for at least 4 hours prior to treatment. Cells were treated with receptor agonists for 2 h and then replaced with fresh full medium and incubated overnight at 37 °C, 5% CO_2_ in a humidified environment. To test the effects of TM peptides (50 μM), AMD3100 (10 μM) or phentolamine (100 μM), cells were pre-incubated with these reagents for 15 min at 37 °C before adding agonists. The following morning, medium was removed from cell culture plates and replaced with a 100 µL 1:5 mixture of Bright-Glo (Promega) and 1× HBSS, 20 mM HEPES solution. Plates were then incubated at room temperature for 20 min before measuring luminescence on a Biotek Synergy II plate reader. Relative luminescence (RLU) is expressed as % of luminescence upon stimulation with the highest concentration of the corresponding receptor agonist when the Tango receptor was co-expressed with pcDNA3 (=100%). When HTLA cells were not transfected with a Tango plasmid, no change in luminescence was detectable upon agonist treatment (Supplementary Fig. S3).

### Immunofluorescence receptor internalization assays

Receptor internalization assays were performed with HEK293T cells and hVSMC. HEK293T cells were transfected with HA-CXCR4 plus pcDNA3 or FLAG- α_1b_-AR at 375 ng each DNA/well of a 12-well plate. After 24 hours, cells were re-plated in poly D-lysine coated 8-well chamber slides. After overnight incubation, cells were chilled on ice and labeled with FITC-conjugated anti-HA (Sigma-Aldrich, H7411, 2 μg/ml in Opti-MEM) for 1 h at 4 °C. Subsequently, labeled cells were washed with Opti-MEM twice and then incubated with CXCL12 (200 nM) or PE (200 μM) for 1 h at room temperature. Thereafter, cells were washed with PBS and fixed with 4% paraformaldehyde. hVSMC were plated in 8-well chamber slides. The next day, slides were cooled on ice, washed once with Opti-MEM and then incubated with FITC-conjugated anti-CXCR4 antibody (R&D, FAB170F, 1:150 dilution in Opti-MEM) or rabbit anti-CXCR4 antibody (Alomone, ACR-014, 1:150 in Opti-MEM) at 4 °C for 1 h. Subsequently, slides were washed twice and then treated with CXCL12 (100 nM) or PE (10 μM) in Opti-MEM at 37 °C for 20 min. Cells were fixed with 4% paraformaldehyde/PBS at room temperature for 10 min. After three washes with PBS, cells incubated with non-conjugated anti-CXCR4 were permeabilized with 0.2% Triton X-100/PBS at room temperature for 10 min and then blocked with 1% BSA/PBS for 30 min, followed by incubation with Alexa 488-conjugated goat anti-rabbit antibody (1:250) at room temperature for 1 h. HEK293T cells and hVSMC Cells were mounted with mounting medium for fluorescence microscopy. Images were obtained under a florescence microscope (Carl Zeiss Axiovert 200 M) with a 40x objective lens equipped with Axio CamMRc5. For each well, 10 vision fields were imaged.

### Chemotaxis assays

Cell migration was assessed using the ChemoTx 96-well cell migration system, as described^17,22,32^. The chemotactic index (CI) was calculated as the ratio of cells that transmigrated through the filter in the presence versus the absence (=PBS/control) of the test solutions.

### Cell viability assays

Cell viability was assessed using the Trypan blue exclusion method. HTLA cells were incubated with various concentrations of phentolamine under the same conditions used in Presto-Tango assays. After staining cells with Trypan blue, at least 100 cells per condition and experiment were examined in a hemocytometer and the percentage of viable cells quantified.

### Data analyses

Data are expressed as mean ± standard error of the mean (SEM) from n independent experiments that were performed on different days. Data were analyzed using the GraphPad-Prism 7 software. Unpaired Student’s t test or one-way analyses of variance (ANOVA) with Dunnett’s multiple comparison post-hoc test for multiple comparisons were used, as appropriate. Dose response curves were generated using non-linear regression analyses. A two-tailed p < 0.05 was considered significant.

### Data availability

The datasets generated during the current study are available from the corresponding author on reasonable request.

## Electronic supplementary material

Supplementary Information
