# Supplementary material for: Asymmetrical ligand-induced cross-regulation of chemokine (C-X-C motif) receptor 4 by α1-adrenergic receptors at the heteromeric receptor complex
Source: Sci Rep. 2018 Feb 9;8:2730. doi: 10.1038/s41598-018-21096-4 (PMC5807542; doi:10.1038/s41598-018-21096-4)
Supplement: Supplementary file 1 — Supplementary Information [file 41598_2018_21096_MOESM1_ESM.pdf]

Supplementary Information

Asymmetrical ligand-induced cross-regulation of chemokine (C-X-C motif) receptor 4 by α_1-adrenergic receptors at the heteromeric receptor complex

Xianlong Gao[1], Lauren J. Albee[1], Brian F. Volkman[2], Vadim Gaponenko[3], and Matthias Majetschak[1/4]

From the [1]Burn and Shock Trauma Research Institute, Department of Surgery, Loyola University Chicago Stritch School of Medicine, Maywood, Illinois 60153, [2]Department of Biochemistry, Medical College of Wisconsin, Milwaukee, Wisconsin 53226, [3]Department of Biochemistry and Molecular Genetics, University of Illinois at Chicago, Chicago, Illinois 60607 and the [4]Department of Molecular Pharmacology and Therapeutics, Loyola University Chicago Stritch School of Medicine, Illinois 60153.

To whom correspondence should be addressed: Matthias Majetschak, Loyola University Chicago Stritch School of Medicine, 2160 S. 1st Avenue, Maywood, IL 60153, Telephone: (708) 327-2472; FAX: (708) 327- 2813; E-mail: mmajetschak@luc.edu

Supplementary Figure S1

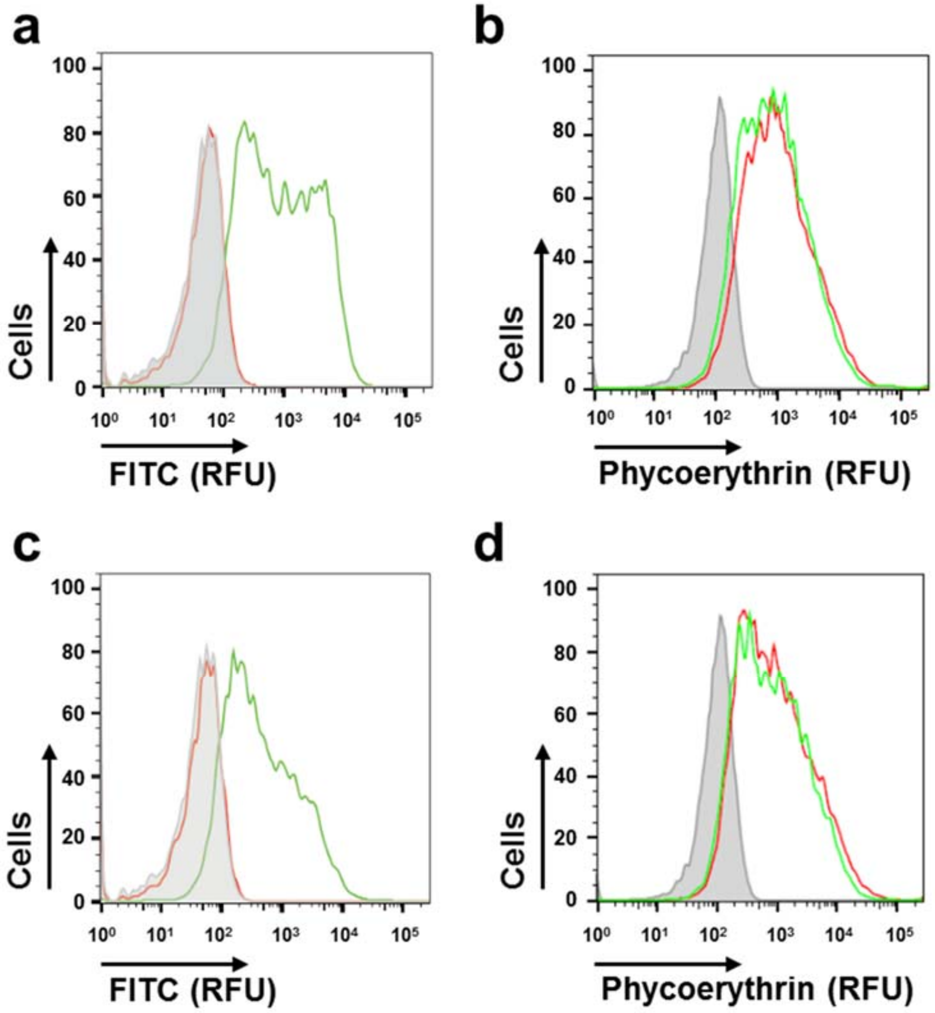

Measurements of receptor expression by flow cytometry. HTLA cells were co-transfected with 0.75 µg DNA encoding FLAG-α_{1b}-AR-TANGO (**a**/**b**) or FLAG-α_{1d}-AR-TANGO (**c**/**d**) plus 0.75 µg pcDNA3 or HA-CXCR4. Grey areas show unstained cells. RFU: relative fluorescence units. Data are representative of n=3 experiments. **a/c.** Measurement of HA-CXCR4 expression. Cells were labeled with FITC-conjugated anti-HA. Red line: cells transfected with FLAG-$\alpha_{1b/d}$-AR-TANGO plus pcDNA3. Green line: cells transfected with FLAG-$\alpha_{1b/d}$-AR-TANGO/HA-CXCR4. **b/d.** Measurement of FLAG-$\alpha_{1b/d}$-AR-Tango expression. Cells were labeled with phycoerythrin-conjugated anti-FLAG. Red line: cells transfected with FLAG-$\alpha_{1b/d}$-AR-TANGO plus pcDNA3. Green line: cells transfected with FLAG-$\alpha_{1b/d}$-AR-TANGO/HA-CXCR4.

Supplementary Figure S2

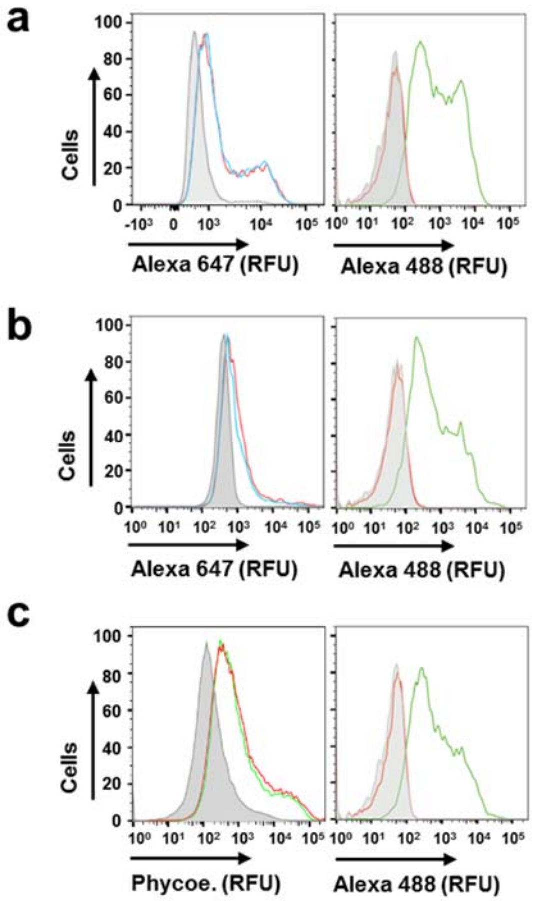

Measurements of receptor expression by flow cytometry. HTLA cells were co-transfected with FLAG-α_{1b}-AR-TANGO plus pcDNA or Myc-CXCR4-TANGO (**a**), FLAG-CXCR4-TANGO plus pcDNA or HA-α_{1b}-AR (**b**) or with FLAG-CXCR4-TANGO plus HA-α_{1b}-AR or Myc-α_{1b}-AR-TANGO or with FLAG-α_{1b}-AR-TANGO plus pcDNA3 or HA-CXCR4 (**c**) (0.75 µg DNA each). RFU: relative fluorescence units. Grey areas show unstained cells. Data are representative of n=3 experiments. **a.** *Left*: Measurement of FLAG-α_{1b}-AR-TANGO cell surface expression. Red line: cells transfected with FLAG-α_{1b}-AR-TANGO/pcDNA3. Blue line: cells transfected with FLAG-α_{1b}-AR-TANGO/CXCR4-Tango. *Right*: Measurement of Myc-CXCR4 cell surface expression. Red line: cells transfected with FLAG-α_{1b}-AR-TANGO/pcDNA3. Green line: cells transfected with FLAG-α_{1b}-AR-TANGO/Myc-CXCR4-TANGO. **b.** *Left*: Measurement of FLAG-CXCR4-TANGO cell surface expression. Red line: cells transfected with FLAG-CXCR4-TANGO/pcDNA3. Blue line: cells transfected with FLAG-CXCR4-TANGO/HA-α_{1b}-AR. *Right*: Measurement of HA-α_{1b}-AR cell surface expression. Red line: cells transfected with FLAG-CXCR4-TANGO/pcDNA3. Green line: cells transfected with FLAG-CXCR4-TANGO/HA-α_{1b}-AR. **c.** *Left*: Measurement of FLAG-CXCR4-TANGO cell surface expression. Red line: cells transfected with FLAG-CXCR4-TANGO/HA-α_{1b}-AR. Green line: cells transfected with FLAG-CXCR4-TANGO/Myc-α_{1b}-AR-TANGO. Phycoe.: Phycoeryhtrin. *Right*: Measurement of Myc-α_{1b}-AR-TANGO cell surface expression. Red line: cells transfected with FLAG-CXCR4-TANGO/HA-α_{1b}-AR. Green line: cells transfected with FLAG-CXCR4-TANGO/Myc-α_{1b}-AR-TANGO.

Supplementary Figure S3

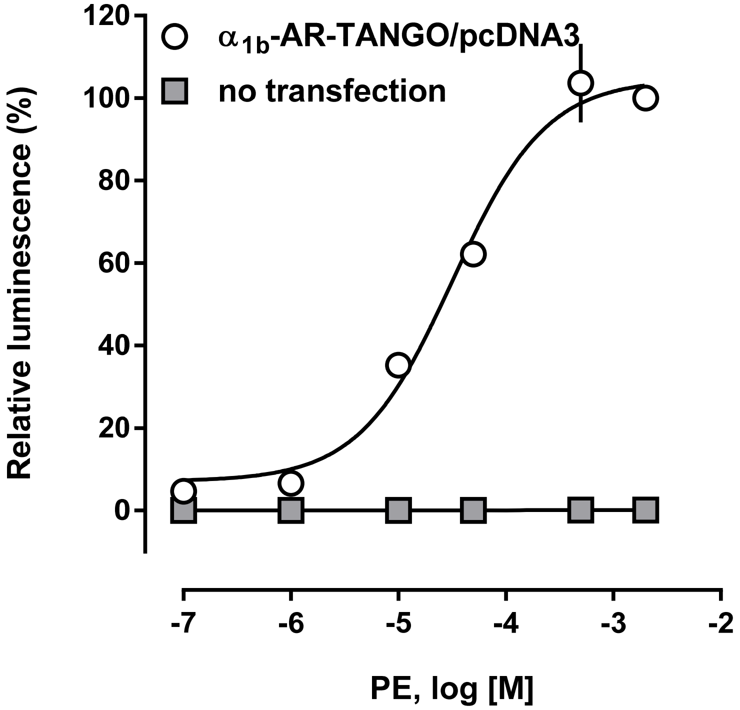

β-arrestin recruitment assay. No change in luminescence is detectable when HTLA cells are stimulated with agonist in the absence of a Tango-GPCR. Non-transfected HTLA cells and HTLA cells transfected with α_{1b}-AR-TANGO plus pcDNA3 were used for β-arrestin 2 recruitment assays. Cells were stimulated with PE (phenylephrine) in triplicate. Data are representative for the PRESTO-Tango assay.
